# 

**Published:** 1832-09

**Authors:** 


### MONTHLY LIST OF MEDICAL BOOKS.

[*Medical Works cannot be entered on this List unless a copy be sent for the purpose, the titles of Books having frequently been sent to us as published which have not been published for weeks, or even months, after.*]

The Cyclopædia of Practical Medicine. Edited by JOHN FORBES, M.D., A. TWEEDIE, M.D., and JOHN CONOLLY, M.D. Part VIII. (published in Monthly Parts,) August 1832. Containing *Expectoration*, by Dr. WILLIAMS; *Favus*, by Dr. A. T. THOMSON; *Feigned Diseases*, by Drs. SCOTT, FORBES, and MARSHALL; *Fever*, by Dr. TWEEDIE; *Fever, Epidemic gastric*, by Dr. CHEYNE; *Fever, Intermittent*, by Dr. BROWN; *Fever, Remittent*, by Dr. BROWN; *Fever, Infantile Remittent*, by Dr. JOY; *Fever, Hectic*, by Dr. BROWN; *Fever, Puerperal*, by Dr. LEE.—Royal 8vo. pp. 127. Sherwood, Gilbert, and Piper; and Baldwin and Cradock, London.

The Anatomy and Physiology of the Organ of Hearing; with Remarks on Congenital Deafness, the Diseases of the Ear, some Imperfections of the Organs of Speech, and the proper Treatment of these several Affections. By DAVID TOD, Member of the Royal College of Surgeons.—8vo. pp. 147. Longman and Co., London.

Clinical Reports of the Surgical Practice of the Glasgow Royal Infirmary. By JOHN MACFARLANE, M.D., Member of the Faculty of Physicians and Surgeons of Glasgow; senior Surgeon to the Royal Infirmary; Lecturer on Clinical Surgery, &c.—8vo. pp. 314. Glasgow; Highley, London.

Part X. The Principles and Practice of Obstetric Medicine, in a Series of Systematic Dissertations on Midwifery, and on the Diseases of Women and Children. Illustrated by numerous Plates. By DAVID D. DAVIS, M.D., M.R.S.S.L., Professor of Midwifery in the University of London, &c.—4to. pp. 24. John Taylor, London.

Essay on the Origin, Symptoms, and Treatment of Cholera Morbus, and of other Epidemic Disorders, with a View to the Improvement of Sanitary Regulations. By T. FORSTER, M.B. F.L.S. M.R.A.S. &c.—8vo. pp. 62. Keating and Brown, London; Treuttell and Wurtz, Paris and Strasbourg.

A Treatise on Cholera; containing the Author's Experience of the Epidemic known by that Name, as it prevailed in the City of Moscow in Autumn 1830 and Winter 1831. By JAMES KEIR, M.D., Professor of Pathology, Therapeutics, and Clinical Medicine, in the Imperial Academy of Medicine and Surgery, &c.—8vo. pp. 138. Black, Edinburgh; and Longman, London.

Practical Observations in Midwifery; with a Selection of Cases. Part II. By JOHN RAMSBOTHAM, M.D., late Lecturer on Midwifery at the London Hospital, &c.—8vo. pp. 507. Highley, London.

We shall, as soon as possible, give an account of this very interesting work.

Observations on Spasmodic Cholera, its Origin, Nature, and Treatment; with Remarks on Epidemic Diseases generally. Second Edition, with Additions and Alterations. By HENRY M'CORMAC, M.D. &c.—8vo. pp. 28. Longman and Co., London.

Experimental Inquiries in Chemical Physiology. Part I. (complete in one volume.) On the Blood; with an Appendix, containing Remarks on the Nature and Treatment of the Cholera Asphyxia. By HORATIO PRATER.—8vo. pp. 304. Highley, London.

### APOTHECARIES' HALL.

**Names of Gentlemen** to whom the Court of Examiners have granted Certificates:

AUGUST 2.

Anthony Allinson, of Willington, Northumb.
Henry Charles Atkinson, Doncaster
William Martin Coates, Salisbury
Thomas Gibson, Wing, Rutlandshire
Charles Underwood, Ross, Herefordshire
William Kelson Wright, Bristol.

AUGUST 9.

Samuel Hayward Ford, of Colchester
William Orton, Narborough
Charles Greville Ruddock, Bridgewater.

AUGUST 16.

James Cochrane, of Edinburgh
Richard King, Tower of London
John Collings Leadbetter, Mirfield, Yorkshire
Charles Mitchell, Surrey
Henry Rees, London
William Smith, Manchester
Henry Waterworth, Isle of Wight
Henry Ward, Dens, Norfolk.

AUGUST 23.

William Arundell Burridge, of Taunton
Robert Walter Crowther, Halifax
Edward Duncan, Newcastle-upon-Tyne
William Henry Gooch, Peckham
Thomas Knowles, Birstall
George Frederick Wilks, Charing, Kent.

TO CORRESPONDENTS.

*** *Communications have been received from* Dr. Webster, Mr. Grant, &c.

